# Relevance of Stereotyped B-Cell Receptors in the Context of the Molecular, Cytogenetic and Clinical Features of Chronic Lymphocytic Leukemia

**DOI:** 10.1371/journal.pone.0024313

**Published:** 2011-08-29

**Authors:** Francesco Maura, Giovanna Cutrona, Sonia Fabris, Monica Colombo, Giacomo Tuana, Luca Agnelli, Serena Matis, Marta Lionetti, Massimo Gentile, Anna Grazia Recchia, Francesco Di Raimondo, Caterina Musolino, Fiorella Ilariucci, Nicola Di Renzo, Emanuela Pesce, Stefano Molica, Massimo Federico, Agostino Cortelezzi, Fortunato Morabito, Manlio Ferrarini, Antonino Neri

**Affiliations:** 1 Ematologia 1 CTMO, Fondazione IRCCS Ca' Granda Ospedale Maggiore Policlinico and Dipartimento di Scienze Mediche, Università di Milano, Milan, Italy; 2 Diagnostica Molecolare, Dipartimento di Tecnologie Diagnostiche Avanzate, Istituto Nazionale per la Ricerca sul Cancro, Genova, Italy; 3 Divisione di Oncologia Medica C, Istituto Nazionale per la Ricerca sul Cancro and Dipartimento di Oncologia, Biologia e Genetica, Università degli Studi di Genova, Genova, Italy; 4 Dipartimento di Oncoematologia, Azienda Ospedaliera di Cosenza, Cosenza, Italy; 5 Sezione di Ematologia, Oncologia e Patologia Generale, Dipartimento di BioMedicina Clinica e Molecolare, Ospedale Ferrarotto, Catania, Italy; 6 Divisione di Ematologia, Università di Messina, Messina, Italy; 7 Unità di Ematologia, Ospedale Santa Maria Nuova, Reggio Emilia, Italy; 8 Unità Operativa di Ematologia e Trapianto di Cellule Staminali, Ospedale Vito Fazzi, Lecce, Italy; 9 Università degli Studi di Modena e Reggio Emilia - Oncologia Medica Policlinico, Modena, Italy; 10 Unità Operativa di Oncologia, Azienda Ospedaliera Pugliese Ciaccio, Catanzaro, Italy; The University of Birmingham, United Kingdom

## Abstract

Highly homologous B-cell receptors, characterized by non-random combinations of immunoglobulin heavy-chain variable (*IGHV*) genes and heavy-chain complementarity determining region-3 (*HCDR3*), are expressed in a recurrent fraction of patients affected by chronic lymphocytic leukemia (CLL). We investigated the *IGHV* status of 1131 productive IG rearrangements from a panel of 1126 CLL patients from a multicenter Italian study group, and correlated the presence and class of HCDR3 stereotyped subsets with the major cytogenetic alterations evaluated by FISH, molecular prognostic factors, and the time to first treatment (TTFT) of patients with early stage disease (Binet A). Stereotyped HCDR3 sequences were found in 357 cases (31.7%), 231 of which (64.7%) were unmutated. In addition to the previously described subsets, 31 new putative stereotypes subsets were identified. Significant associations between different stereotyped *HCDR3* sequences and molecular prognostic factors, such as CD38 and ZAP-70 expression, *IGHV* mutational status and genomic abnormalities were found. In particular, deletion of 17p13 was significantly represented in stereotype subset #1. Notably, subset #1 was significantly correlated with a substantially reduced TTFT compared to other CLL groups showing unmutated *IGHV*, ZAP-70 or CD38 positivity and unfavorable cytogenetic lesions including del(17)(p13). Moreover, subset #2 was strongly associated with deletion of 13q14, subsets #8 and #10 with trisomy 12, whereas subset #4 was characterized by the prevalent absence of the common cytogenetic abnormalities. Our data from a large and representative panel of CLL patients indicate that particular stereotyped *HCDR3* sequences are associated with specific cytogenetic lesions and a distinct clinical outcome.

## Introduction

Chronic lymphocytic leukemia (CLL) is a common disorder characterized by the monoclonal accumulation of B lymphocytes with a distinct phenotype (CD5-positive, CD23-positive, CD22-negative and low level of surface Ig) and a highly variable clinical course [1]–[3]. A different clinical outcome has been associated with peculiar cellular and molecular markers and/or specific genomic alterations [4]–[6]. In particular, the mutational status of the immunoglobulin heavy-chain variable (*IGHV*) gene defines two disease subgroups; one subgroup is characterized by the absence of somatic mutation in CLL cells and has the worst clinical course and outcome, whereas the other, with somatic mutations in *IGHV* genes, has a more benign prognosis and outcome [7], [8]. A biased repertoire of *IGHV*-diversity (*D*)- joining (*J*) genes has been reported to characterize the B cell receptor (BCR) in CLL, with a different prevalence of certain genes in the mutated (M) or unmutated (UM) group, respectively [9]. Moreover, more than 20% of CLL patients exhibit closely homologous (“stereotyped”) heavy chain complementary-determining region 3 (*HCDR3*) sequences and approximately 1% of these also carry virtually identical IGHV amino-acid sequences [10]–[13]. These findings have suggested that clones sharing stereotyped BCRs may expand because of stimulation by a restricted set of epitopes and that antigenic driving may play an important role in the pathogenesis of the disease [6], [14]–[16].

Although recent data have suggested the existence of specific correlation between stereotyped subsets and common cytogenetic lesions [17], [18] or clinical outcome [10], [13], [19], [20], it remains to be defined whether the expression of distinct BCRs in CLL may be relevant to the molecular and cytogenetic profile and/or to the clinical outcome in at least a fraction of patients.

In the present study, we investigated the BCR repertoire in 1126 CLL patients recruited by a multicenter Italian study group. Based on previously reported criteria [10], [21] and canonical sequence alignment procedures, we searched for the known stereotyped subsets in three publicly available data sets [10], [22], [23], as well as for potential novel subsets by performing a pair-wise alignment in the proprietary dataset. The most represented stereotyped subsets were then investigated for their association with the common molecular and cytogenetic features as well as for their impact on clinical outcome of early stage patients (Binet A).

## Methods

### Patient samples

Written informed consent was obtained from all patients in accordance with the declaration of Helsinki and the study was approved by the local Ethics Review Committee (Comitato Etico Provinciale, Modena, Italy). All patients were diagnosed according to the National Cancer Institute Working Group criteria [24]. Our dataset counted a total of 1126 CLL patients with productive *IGHV-D-J* rearrangement included in retrospective (745 patients) and prospective (381 patients, O-CLL1-GISL protocol) multicenter Italian studies from all over the country. In all cases genomic, cytogenetic and molecular analyses were performed on highly purified peripheral mononuclear B-cells from blood samples collected within one year of diagnosis, provided that the patient remained untreated.

### Molecular and FISH analyses

CLL *IGHV* gene usage and mutation were determined as previously described and the 98% homology cut-off value was used to discriminate the M or UM *IGHV* configuration [9]. ZAP-70 and CD38 expression were investigated by immunophenotypic analysis as previously described [25]–[27]. Specifically, a cut-off ≥20% or ≥30% positive cells was chosen to discriminate ZAP-70 or CD38 positive from negative patients. Cytogenetic abnormalities involving deletions at chromosomes 11q23, 13q14 and 17p13 and trisomy of chromosome 12 were investigated by fluorescence in situ hybridization (FISH) as previously described [28]. FISH analyses were performed in all of the patients for whom biological material was available, and no prior selection based on age or disease progression was applied. Time to First Treatment (TTFT) was defined as time from diagnosis to first line treatment (event) or to last follow-up (censored observation). Treatment was decided uniformly in all participating centers based on documented progressive and symptomatic disease according to NCI working guidelines [24]. TTFT information was available for 739 patients (661 staged as Binet A; 56 as Binet B and 22 as Binet C), median follow up was 30 months (range 1–316 months), and 237 (32.1%) patients had received treatment by the end of the follow up.

### Identification of stereotyped subsets and statistical analysis

We assigned a stereotyped cluster label to our HCDR3 sequences by means of pair-wise alignment with known stereotyped sequences available from different public databases [10], [22], [23]. In concordance with previously proposed methods, we applied a primary filter excluding pairs of sequences whose length differed more than 3 amino acids and we considered as stereotyped those sequences sharing more than 60% identity on alignments showing less than 3 gaps [10], [29]. Such analysis was performed using a *global alignment algorithm* [30] with BLOSUM62 as the similarity matrix [31] under the BioStrings package for *Bioconductor*. The same approach was applied to discover new potential stereotyped clusters with pair-wise alignments of the sequences from the proprietary database. A supplemental “GX” number was assigned to novel putative subsets not previously included in the Murray *et al* and Bomben *et al* nomenclature system [22], [23]. All contingency analyses were performed by Fisher's Exact test. The competing effect of death on the relationship between TTFT and stereotyped BCRs was modeled by proportional hazards of competing risks. Correlation with TTFT was tested between the considered groups in Binet A patients using the *crr* function of *cmprsk* package in R software [32]. A *P* value <0.05 was considered significant for all statistical calculations. All data were statistically analyzed using conventional procedures available in R (www.r-project.org).

## Results

A total of 1126 CLL patients were investigated for productive *IGHV-D-J* rearranged sequences; 5 patients carried a double in frame productive rearrangement. Based on the 98% homology criteria, 405/1126 patients (36%) were classified as UM (Table 1 and Figure S1). *IGHV*, *IGHD* and *IGHJ* gene type and distribution are reported in Figure S2A–C [10], [22], [23]. ZAP-70 and CD38 expression were positive in 367/1011 (36.3%) and 306/1051 (29.1%%) of cases, respectively. Interphase FISH was performed on 704 patients and at least one abnormality was found in 466/704 (66.2%) of cases. Based on proposed hierarchical classification [4]–[6], del(13)(q14) was found as the sole abnormality in 287/704 (40.7%); trisomy 12 was found in 98/704 (13.9%) patients and associated with del(13)(q14) in 9 cases; del(11)(q23) was found in 46/704 (6.5%) patients and associated with del(13)(q14) and trisomy 12 in 25 and 1 cases, respectively; del(17)(p13) was found in 35/704 (5%) patients and associated in 5 and in 1 cases with del(13)(q14) and trisomy 12, respectively.

**Table 1:** Biological, molecular and cytogenetic features of CLL patients included in the study.

Variable	N° of positive/investigated patients (%)	Correlation with TTFT
**ZAP-70**	367/1011 (36.3%)	*P<*0.0001
**CD38**	306/1051 (29.1%)	*P*<0.0001
***IGHV*** *	405/1126 (36%)	*P*<0.0001
**No FISH alteration** **	238/704 (33.8%)	*P*<0.0001
**Del(13)(q14)**	287/704 (40.7%)	
**Trisomy 12**	98/704 (13.9%)	
**Del(11)(q23)**	46/704 (6.5%)	
**Del(17)(p13)**	35/704 (5%)	

* the number of investigated sequences was 1131 (see text).

** FISH results according to the hierarchical classification [4]–[6].

### Identification of stereotyped sequences

To identify stereotyped *HCDR3* sequences occurring in our dataset, we performed a global alignment analysis which allowed (*a*) to compare each of our cases with publicly available data [10], [22], [23] and (*b*) to investigate the occurrence of new putative stereotypes within the proprietary database. Using this approach, stereotyped sequences were found in 357/1126 (31.7%) of the patients, 64.7% (231/357) of which were UM (*P*<0.0001), further supporting previous evidence [10], [22], [23].

Among patients with stereotyped *HCDR3*, 294 (82.3%) belonged to previously described subsets. In particular, the most recurrent subsets identified in our study were #1 (32 pts), #4 (29 pts), #7 (22 pts), #2 (20 pts), #3 (16 pts) and #9 (15 pts) (Table S1). Of note, the global alignment procedures performed on pair-wise sequences of our dataset allowed the identification of 31 new putative subsets in 63 patients (63/357;17.7%) recorded with a progressive code from G1 to G31 (see Table S2).

### Correlation between cellular and molecular features with stereotyped BCRs in CLL

We then evaluated the prevalence of known molecular, biological and cytogenetic markers in the most represented and characterized stereotyped subsets of our dataset (Table 2). Subset #1 (*IGHV1*-5-7/*IGHD6*-19/*IGHJ4*) was the most frequent in our cohort. Despite the relatively heterogeneous gene usage, it was associated with UM *IGHV* genes in all of the cases (32pts). Subset #1 patients were more frequently ZAP-70 and CD38 positive compared to all the other patients (*P*<0.0001) or those utilizing the same *IGHV* genes without stereotyped *HCDR3* (*P* = 0.0002 and *P*<0.0001 for ZAP-70 and CD38, respectively). However, no significant association between subset #1 and ZAP-70/CD38 positivity was identified when compared to patients with the UM *IGHV* configuration (data not shown). Considering only patients (18/32;56.3%) evaluated by FISH, we observed a higher prevalence of unfavorable deletions (7/18; 38.9%), particularly del(17)(p13) (5/18; 27.8%) (Table 2). Notably, the prevalence of del(17p)(p13) in subset #1 patients was significantly higher than that found in all the remaining patients (30/686;4.3%) (*P* = 0.0012). To avoid that the prevalent UM status in subset #1 may represent a bias factor, we compared the frequency of del(17)(p13) between subset #1 and all the remaining UM patients (22/244; 9%) confirming the previous association (*P* = 0.0266). Furthermore, comparing subset #1 patients with those showing the same *IGHV* gene usage without homologous *HCDR3*, we observed that del(17)(p13) retained its significant correlation (*P* = 0.0064). The percentage (median 78%: range 33.5–99) of malignant cells carrying the del(17)(p13) in subset #1 patients did not differ significantly in the remaining ones (30pts) having the del(17)(p13) (data not shown).

**Table 2:** The molecular, cytogenetic and clinical characterization of the most representative and described subsets.

Subset	N° of pts	Binet A*	UM	CD38	ZAP-70	FISH-neg	del(13)	12	del(11)	del(11) and del(13)	del(17)	del(17) and del(13)
***#1***	32	13	32/32	21/32	22/30	4/18	2/18	5/18	2/18	0/18	3/18	2/18
***#2***	20	12	10/20	3/17	5/15	1/13	10/13	0/13	0/13	2/13	0/13	0/13
***#3***	16	8	16/16	6/14	10/13	6/11	0/11	3/11	0/11	0/11	2/11	0/11
***#4***	29	19	1/29	0/25	3/24	13/23	9/23	1/23	0/23	0/23	0/23	0/23
***#5***	5	2	5/5	3/4	2/4	0/2	0/2	1/2	0/2	1/2	0/2	0/2
***#6***	5	2	5/5	3/3	1/3	2/4	1/4	0/4	0/4	0/4	1/4	0/4
***#7***	22	10	22/22	12/21	16/21	4/13	4/13	1/13	2/13	2/13	0/13	0/13
***#8***	8	5	8/8	6/7	7/7	1/7	0/7	5/7	0/7	0/7	1/7	0/7
***#9***	15	7	15/15	6/14	6/10	1/9	2/9	2/9	2/9	1/9	1/9	0/9
***#10***	11	5	10/10	8/10	10/10	1/7	0/7	6/7	0/7	0/7	0/7	0/7

*Patients in Binet A with complete follow-up.

A number of additional correlations between distinct stereotyped *HCDR3* and cytogenetic/molecular features were evidenced in our panel. The cytogenetic profile of subset #2 (*IGHV3*-21) was characterized by a very strong prevalence of del(13)(q14) (12/13; 92.3%), found as the sole abnormality in 10 patients and associated with the del(11)(q23) in the remaining two (Table 2). When compared to the whole panel (313/684; 45.7%), this finding was statistically significant (*P* = 0.0009). Of note, differently from another group [13] we did not observe any significant difference between patients admitted to Institutions from North or South Italy in terms of *IGHV3*-21 usage (20/548 and 14/578 respectively) or subset #2 (13 and 6, respectively) prevalence (Table S3).

Subset #4 (*IGHV4*-34) was characterized by an almost complete M *IGHV* configuration and negative ZAP-70/CD38 expression (Table 2). In addition, we found that subset #4 patients were characterized by a low incidence of genomic aberrations. In fact, among the 23 cases investigated by FISH, 13 (56.5%) were negative for the most common cytogenetic lesions; this finding was statistically significant when compared to all patients (225/681; *P* = 0.02) or those with M *IGHV* configuration (142/420; *P* = 0.0408). Trisomy 12 was strongly associated with both subset #8 (*IGHV4*-39/*IGHD6*-13/*IGHJ5*) (5/7; 71.4%) and subset #10 (*IGHV4*-39 and *IGHV2*-5/*IGHD2*-2/*IGHJ6*) (6/7; 85.7%), confirming its higher prevalence in *IGHV4*-39 stereotyped patients [17], [20]. Specifically, trisomy 12 was significantly associated with subset #8 and #10 either when all patients or only those with UM *IGHV* configuration were considered (data not showed). Finally, subsets #8 and #10 were strongly associated with ZAP-70 positivity when all patients were considered (*P* = 0.0008 and *P*<0.0001 respectively). Instead, only subset #10 retained its significant association with ZAP-70 considering only UM patients (*P* = 0.017). Moreover, a significant association between subset #8 and #10 and CD38 positivity was found when compared to all patients (*P* = 0.003 and *P* = 0.0013 respectively), but not to UM patients.

### Clinical relevance of stereotyped subsets

**Figure 1:**
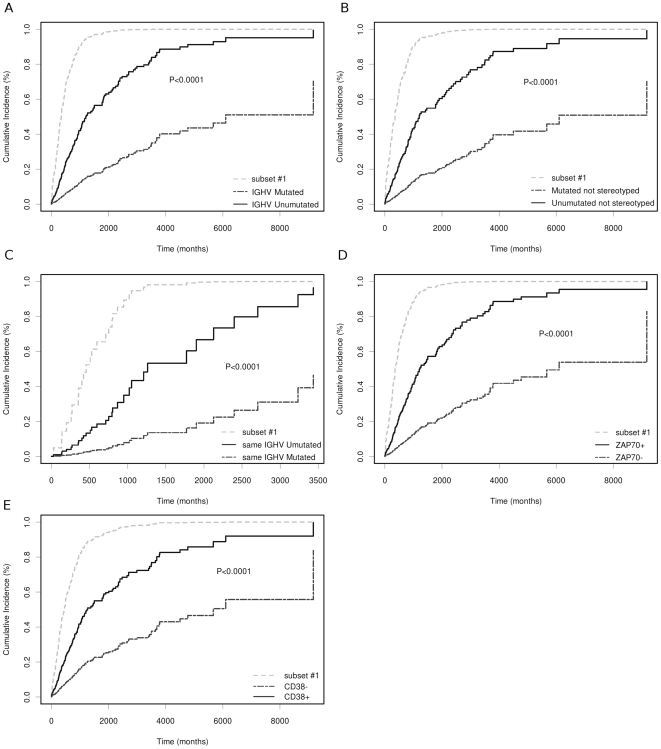
TTFT analysis of subset #1. **A**: Subset #1 patients compared with all UM and M patients. **B**: Subset #1 compared with all non-stereotyped UM and non-stereotyped M patients. **C**: Subset #1 compared with all UM and M patients showing the same *IGHV* usage of subset #1. **D** and **E**: Subset #1 patients compared with the remaining cases grouped according to ZAP-70 and CD38 expression positivity, respectively.

**Figure 2:**
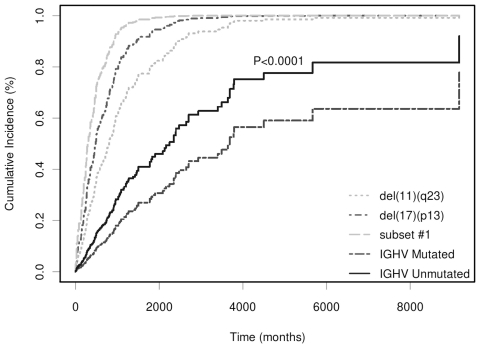
TTFT analysis of subset #1. Subset #1 patients were compared to those positive for del(17)(13p) and del(11)(q23), and those with UM or M *IGHV* status.

We investigated whether the stereotype configuration was correlated with disease progression. For this purpose only Binet A patients were considered. Subset #1 (13 patients, see Table 2) exhibited a significantly reduced TTFT when compared to all UM patients (*P*<0.0001), UM non-stereotyped patients (*P*<0.0001), or UM patients with the same *IGHV* gene usage (*P*<0.0001) (Figure 1A–C). Additionally, subset #1 was associated with an increased risk of earlier treatment compared to the presence of ZAP-70 and CD38 positivity (*P*<0.0001) (Figure 1D–E). The clinical course of subset #1 patients appeared to be similar to that of patients with del(17p) or del(11q) (Figure 2). A multivariate analysis using a proportional hazard model showed that subset #1 retained independence (*P* = 0.0132) from del(17p), del(11q), *IGHV* configuration and ZAP-70 or CD38 positivity. Moreover, considering only UM patients, the prognostic power of subset #1 maintained its significant independence from ZAP-70, del(17p), del(11q) and CD38 (*P* = 0.0102).

In agreement with previous reports [13], [19], [22], subset #2 (*IGHV3*-21) stereotyped patients had an unfavorable clinical course compared to all other patients (*P* = 0.00033) or to those utilizing non-stereotyped *IGHV3*-21 sequences (*P* = 0.052). In addition, subset #2 did not show a more unfavorable clinical course compared to UM patients (*P*>0.05).

Patients in subset #4 were characterized by a long TTT. In fact, only 2/19 patients in Binet A had been treated at the time of the study: of these, one was highly positive for ZAP-70 and the other was the only one with UM *IGHV* configuration.

Finally, likely due to the low number of cases in our series, we were unable to confirm the previously described [10] favorable or unfavorable clinical outcome of subset #5 (*IGHV1*-69/*IGHD3*-10/*IGHJ6*) or subset #3 (*IGHV1*-69/*IGHD2*-2/*IGHJ6*), respectively.

## Discussion

In order to contribute to the elucidation of *HCDR3* stereotyping in CLL, we characterized the BCR repertoire in a comprehensive panel of 1126 patients with the following aims: (*a*) to investigate whether *HCDR3* stereotyped sequences might be correlated with molecular and cytogenetic profiles; and (*b*) to evaluate the putative clinical relevance in terms of TTFT for the most represented stereotypes.

In our study, we identified a total of 31.7% stereotyped *HCDR3* sequences using an amino acid sequence alignment approach according to previously reported criteria [10]. This percentage was slightly higher than those reported to date [10], [22], [23], a finding in all likelihood related to the assessment of proprietary database stereotypes against previously reported ones. In fact, limiting the analysis only to patients included in our cohort, the percentage decreased to 28.3%, as 31 novel putative stereotyped sequences were identified after comparison with the published registries. Therefore, such a procedure (auto-matching and matching with published data) may represent an optimal and unbiased approach to perform stereotyped BCR characterization in CLL.

Our study revealed that subset #1, known to be the most frequent (9% of all stereotyped cases and 7.9% of all UM patients in our series) and characterized by UM *IGHV* configurations, was significantly associated with del(17)(p13). Notably, subset #1 exhibited a more unfavorable clinical course than other patients with an UM *IGHV* configuration, independently of the presence of other adverse prognostic factors, such as del(17)(p13), del(11)(q23), ZAP-70 and CD38 positivity or the usage of *IGHV* genes. The finding that subset #1 shows the worst clinical outcome as found in patients exhibiting 11q23 or 17p13 deletion suggests that it might represent a reliable marker for high risk CLLs in the early stage of the disease.

As regards subset #2 (*IGHV3*-21*),* we confirmed its more unfavorable clinical outcome. However, differently from previously reported data [13], we did not observe a significant difference in the geographical distribution of *IGHV3*-21 across Italy in our cohort of patients. Moreover, we found the presence of del(13)(q14) in virtually all patients tested by FISH (12/13); this finding is in accordance with data recently published by Marincevic *et al* [18], suggesting that this association could be considered *subset-specific*. In addition, we did not observe a strong association between del(11)(q23) and subset #2 as described by the same authors [18]. This discrepancy could be partially explained by the lower number of subset #2 patients analyzed by FISH in our study. However, it should be noted that all but one of the subset #2 cases in our panel were Binet A, whereas 70% of those from Marincevic *et al*. [18] were either in advanced clinical stages or no information was provided, thus preventing any definitive comparison.

In our study, we described a recurrent favorable cytogenetic profile and the indolent course in subset #4 patients. This finding is in agreement with data reported by some authors [10], [18], but not by others [17], leaving this aspect still controversial. Finally, we confirmed that trisomy 12 was correlated with *IGHV4*-39 stereotyped HCDR3 subsets #8 and #10 [17], [20] showing that these two subsets were particularly associated with higher CD38 expression.

In conclusion, our study indicates that distinct stereotyped *HCDR3* regions of BCR in CLL are characterized by specific cytogenetic and/or molecular profiles and clinical course. Further validation in larger and prospective series of patients may help to better clarify distinct biological and clinical features of specific stereotyped subsets.

## 

## Supporting Information

Figure S1 **Predictive value of Binet A,** ***IGHV*** **gene status, CD38, ZAP-70 and the most common genomic aberrations evaluated by FISH.** Cases were subdivided according to Binet classification (A), CD38 expression (B), FISH (C), *IGHV* gene status (D) and ZAP-70 expression (E) before determining TTFT.
(TIF)

Figure S2 **(A)** ***IGHV*** **distribution and association with mutated (M) or unmutated (UM)** ***IGHV*** **configuration.** *IGHV* bars were ordered according to the total number of patients belonging to each subset. Among the most represented *IGHV* genes, there was a higher prevalence of M configuration in *IGHV3*-23 (84/95; 88.4%), of *IGHV4-34* (97/108; 89.8%), (73/83; 87.9%) of *IGHV3-7* (73/83; 87.9%) and of IGHV3-30 (49/66; 74.2%) cases (representing 42% of all M patients); conversely, 91.4% (106/116) of *IGHV1*-69 patients were UM (representing 26.1% of all UM CLL gene usage). The *IGHV3*-21 gene was present in only a small fraction of cases of our panel (34/1126, 3%; 14 UM and 20 M), confirming its low prevalence in a Mediterranean cohort of CLL patients. (**B**) *IGHD* distribution and association with M and UM *IGHV* configuration. *IGHD* bars were ordered by the total number of patients belonging to each subset. *IGHD* gene distribution was similar to that described for other cohorts. *IGHD3*-3 was the most used *IGHD* gene and it was significantly associated with the UM configuration (108/133; 81.2%). On the contrary, *IGHD3*-10 (67/95; 70.5%), *IGHD2*-15 (43/49; 87.8%), *IGHD1*-26 (50/59; 54.7%), and *IGHD3*-22 (64/97; 66%) were significantly associated with the M configuration. (**C**) *IGHJ* gene distribution and association with *IGHV* mutational status. *IGHJ4* and 6 were the most represented *IGHJ* gene and they were associated with M (361/498; 72.5%) and UM (189/353; 53.5%) mutational status, respectively.
(TIF)

Table S1 **BCR molecular features of previously described subets.**
(XLS)

Table S2 **Clinical and molecular features of 31 new putative subsets**
(XLS)

Table S3 **Distribution of IGHV3-21 in patients from North and South Italy.**
(XLS)
