# Supplementary figures and images for: Relevance of Stereotyped B-Cell Receptors in the Context of the Molecular, Cytogenetic and Clinical Features of Chronic Lymphocytic Leukemia
Source: PLoS One. 2011 Aug 29;6(8):e24313. doi: 10.1371/journal.pone.0024313 (PMC3163661; doi:10.1371/journal.pone.0024313)

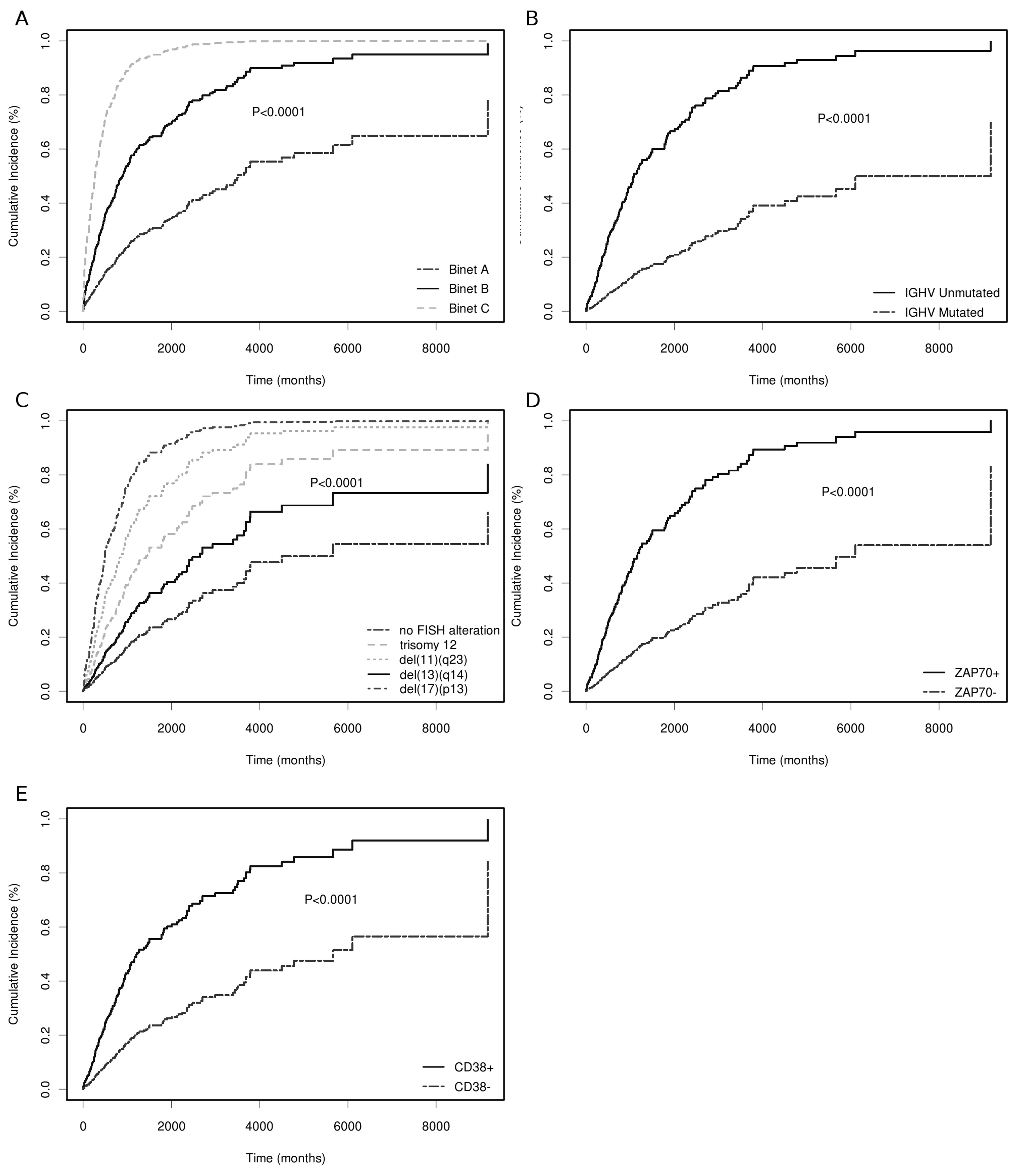

Supplement: Figure S1 — Predictive value of Binet A, IGHV gene status, CD38, ZAP-70 and the most common genomic aberrations evaluated by FISH. Cases were subdivided according to Binet classification (A), CD38 expression (B), FISH (C), IGHV gene status (D) and ZAP-70 expression (E) before determining TTFT. (TIF) [file pone.0024313.s001.tif]

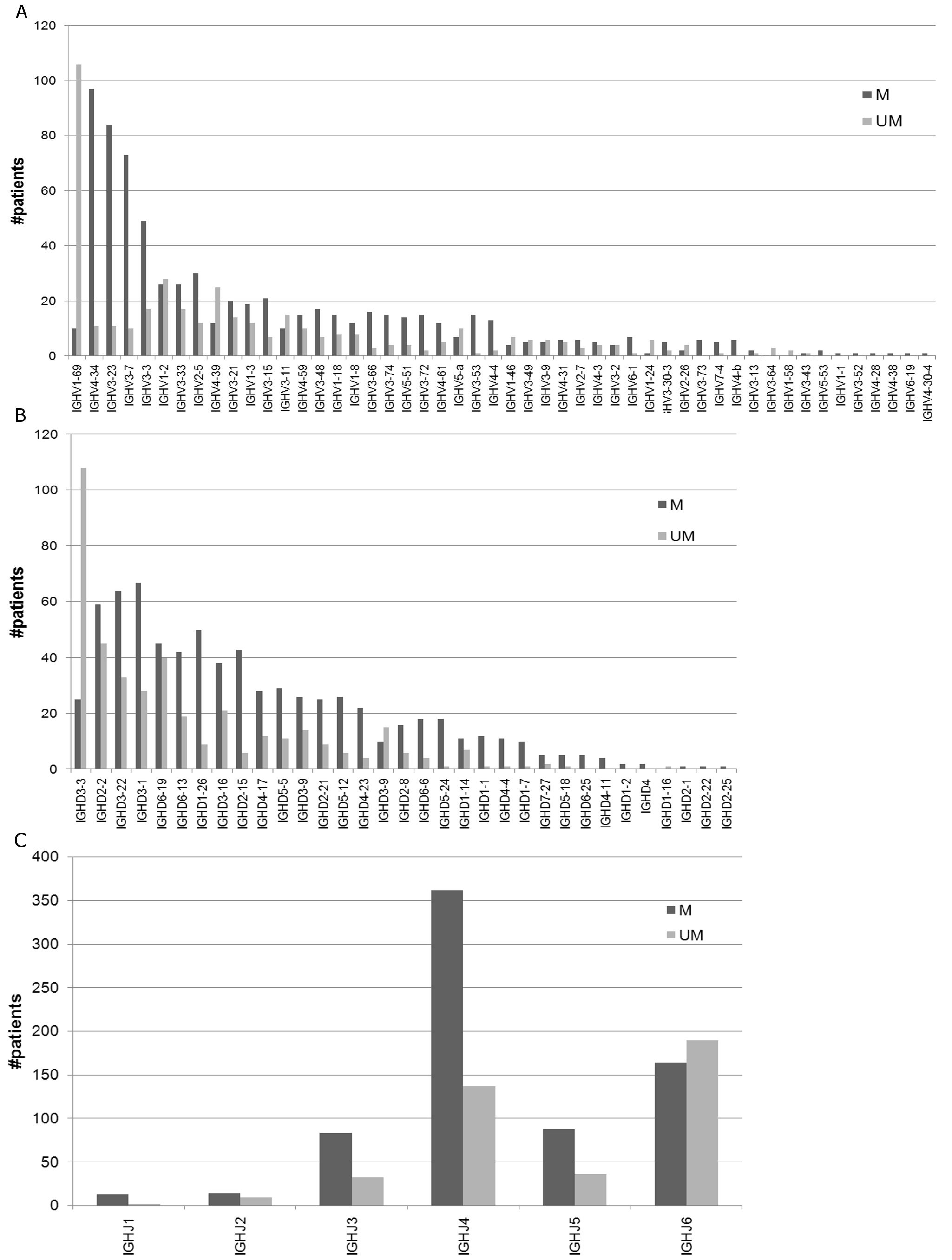

Supplement: Figure S2 — (A) IGHV distribution and association with mutated (M) or unmutated (UM) IGHV configuration. IGHV bars were ordered according to the total number of patients belonging to each subset. Among the most represented IGHV genes, there was a higher prevalence of M configuration in IGHV3-23 (84/95; 88.4%), of IGHV4-34 (97/108; 89.8%), (73/83; 87.9%) of IGHV3-7 (73/83; 87.9%) and of IGHV3-30 (49/66; 74.2%) cases (representing 42% of all M patients); conversely, 91.4% (106/116) of IGHV1-69 patients were UM (representing 26.1% of all UM CLL gene usage). The IGHV3-21 gene was present in only a small fraction of cases of our panel (34/1126, 3%; 14 UM and 20 M), confirming its low prevalence in a Mediterranean cohort of CLL patients. (B) IGHD distribution and association with M and UM IGHV configuration. IGHD bars were ordered by the total number of patients belonging to each subset. IGHD gene distribution was similar to that described for other cohorts. IGHD3-3 was the most used IGHD gene and it was significantly associated with the UM configuration (108/133; 81.2%). On the contrary, IGHD3-10 (67/95; 70.5%), IGHD2-15 (43/49; 87.8%), IGHD1-26 (50/59; 54.7%), and IGHD3-22 (64/97; 66%) were significantly associated with the M configuration. (C) IGHJ gene distribution and association with IGHV mutational status. IGHJ4 and 6 were the most represented IGHJ gene and they were associated with M (361/498; 72.5%) and UM (189/353; 53.5%) mutational status, respectively. (TIF) [file pone.0024313.s002.tif]
